# Critical COVID-19 disease: Clinical course and rehabilitation of neurological deficits

**DOI:** 10.3389/fneur.2022.1012685

**Published:** 2022-10-28

**Authors:** Corinna Wimmer, Marion Egger, Jeannine Bergmann, Volker Huge, Friedemann Müller, Klaus Jahn

**Affiliations:** ^1^Department of Neurology and Intensive Care Medicine, Schoen Clinic Bad Aibling, Bad Aibling, Germany; ^2^German Center for Vertigo and Balance Disorders, Ludwig-Maximilians University (LMU), Munich, Germany; ^3^Pettenkofer School of Public Health, Institute for Medical Information Processing, Biometry, and Epidemiology, Ludwig-Maximilians University (LMU), Munich, Germany

**Keywords:** COVID-19, SARS-CoV-2, neurological rehabilitation, critical care outcomes, neurological manifestations

## Abstract

**Background:**

The COVID-19 disease frequently causes neurological symptoms. Critically ill patients often require neurorehabilitation for manifestations like intensive care unit (ICU) acquired weakness or encephalopathy. The outcome of these patients, however, is largely unknown. Here we report the clinical course of critical affected COVID-19 patients from hospital admission to discharge from inpatient neurorehabilitation.

**Methods:**

Prospective cohort study. COVID-19 patients admitted to neurorehabilitation were included based on a laboratory-confirmed SARS-CoV-2 infection. Assessments [modified Rankin Scale (mRS), Barthel-Index, Fatigue-Severity-Scale-7 and health-related quality of life (EQ-5D-5L)] were conducted at admission and before discharge from inpatient care. Data were compared to the preclinical health status.

**Results:**

Sixty-one patients (62 ± 13 years, 16 female) were included in the analysis. Most patients had been treated on ICU (*n* = 58; 57 ± 23 days) and had received invasive ventilation (*n* = 57; 46 ± 21 days). After discharge from ICU, patients spent on average 57 ± 26 days in neurorehabilitation. The most frequent neurological diagnoses were ICU-acquired weakness (*n* = 56) and encephalopathy (*n* = 23). During rehabilitation overall disability improved [mRS median (IQR) 4.0 (1.0) at inclusion and 2.0 (1.0) at discharge]. However, the preclinical health state [mRS 0.0 (0.0)] was not regained (*p* < 0.001). This was also reflected by the Barthel-Index [preclinical 100.0 (0.0), at inclusion 42.5 (35.0), at discharge 65.0 (7.5); *p* < 0.001]. Patients had only minor fatigue during inpatient care. Quality of life generally improved but was still low at discharge from hospital.

**Conclusion:**

Patients with neurological sequelae after critical COVID-19 disease showed substantial deficits at discharge from inpatient care up to 4 months after the initial infection. They were restricted in activities of daily living and had reduced health-related quality of life. All patients needed continued medical support and physical treatment.

## Introduction

The Corona pandemic has resulted in millions of infections with SARS-CoV-2 worldwide and continues to cause numerous new infections. By August 2022, more than 6.4 million people had died in association with SARS-CoV-2 infection (https://covid19.who.int/, as of August 04, 2022). Although many patients recover within days to weeks, a substantial proportion develops long-standing symptoms (long-COVID) ranging from mild fatigue and reduced physical fitness to immobility and long-term disability. The presumed number of patients affected by long-COVID ranges from 2.3 to 53.0% in the current literature (1). Different parameters, such as multiple organ involvement during the acute phase of the disease, persistent reservoirs of the virus in different tissues, as well as manifestation in the central nervous system and immune system dysregulation, are hypothesized to contribute to symptom persistence (2, 3).

Neurological and neuropsychiatric complications of SARS-CoV-2 infections frequently occur, with the utmost prevalence reported for anosmia (43.1%), muscular weakness (40.0%), and fatigue (37.8%). Other common neurologic or neuropsychiatric symptoms include headache, dysgeusia, myalgia, depression as well as sleep disorder (4). Within a study including nearly 5,000 hospitalized patients with COVID-19, a 38% increased hazard of in-hospital death and a decreased likelihood of discharge home among patients diagnosed with a neurological disorder was reported (5). The pooled prevalences of severe complications such as stroke (2%) and encephalopathy (7%) might be lower (6), however, they can cause substantial disability and often trigger neurorehabilitation. Furthermore, patients with critical COVID-19 disease and prolonged invasive ventilation are at high risk of developing neuromuscular weakness. This was shown in an observational study in 110 critically ill patients with COVID-19 treated in the intensive care unit (ICU). All patients showed ICU-acquired weakness (ICUAW) on awakening (7). In another study with patients requiring intubation due to COVID-19, neurologic outcomes were investigated 3 and 6 months after ICU discharge. Cognitive impairment, muscle weakness, and psychologic symptoms were frequent and 74% still required follow-up interventions like physiotherapy or neuropsychological therapy (8). These cases emphasize the necessity of neurorehabilitation in most cases (9, 10). However, studies about rehabilitation after COVID-19 often focused on pulmonary rehabilitation, reported on rehabilitation in less severely affected patients, investigated therapies in patients during the acute phase or with community-dwelling participants or outpatients (11–15).

The rehabilitation of critically ill patients after COVID-19 with neurologic symptoms has not yet been described. Therefore, the objective of this study was to describe the clinical course of critically ill patients with COVID-19 and neurological sequelae during inpatient neurorehabilitation. We hypothesized that patients would show incomplete recovery and that disability and fatigue at discharge can be predicted by the severity of the disease, i.e., the length of stay on ICU.

## Methods

### Study population

Patients for this prospective cohort study were recruited at one of the largest neurorehabilitation centers in Germany (Schoen Clinic Bad Aibling). Adult patients (≥18 years) with laboratory-confirmed COVID-19 (nasal or pharyngeal swab for SARS-CoV-2, evaluated by real-time reverse transcriptase-polymerase chain reaction (PCR) assay) were included after being tested non-infectious (two negative PCR-tests) and discharged from the ICU. Exclusion criteria were insufficient communication skills (that would interfere with the accomplishment of the questionnaires and assessments) and patients treated with a main diagnosis different than COVID-19 during hospitalization. Data represent the interim analysis of an ongoing larger cohort study with follow-up assessments up to 1 year after hospital discharge. Patients who completed the first two study visits (at study inclusion and at hospital discharge) are entailed in this analysis. All patients received at least 100 min per day of neurorehabilitation therapies including physiotherapy (gait rehabilitation, aerobic, endurance and resistance training, balance training, physical therapy etc.), occupational therapy (training of gross and fine motor skills of the upper extremities, training of activities of daily living like grooming, dressing, using the toilet, resistance training, treatment of sensory deficits etc.) dysphagia therapy, respiratory therapy and neuropsychology (therapy for deficits of attention, concentration, processing speed, memory and executive functions, supportive conversations for coping with the illness and relaxation training). Treatment distribution was tailored to individual patient necessities and therapies were conducted in single or group settings.

### Standard protocol approvals, registrations, and patient consents

The study was approved by the medical ethics committee of the Ludwig-Maximilians University of Munich (project no. 20-0478) and the study conforms with the World Medical Association Declaration of Helsinki. Written informed consent was obtained from all participants (or their legal guardians). The study was registered at the German Clinical Trials Register (No. DRKS00025606).

### Procedures, scales and scores

Disease severity was categorized by the Seven-Category Ordinal Scale (16). The first two categories describe patients not being hospitalized, categories three to six comprise patients being hospitalized with increasing disease severity (e.g., need for non-invasive mechanical ventilation) and the seventh category includes death.

During rehabilitation, two study visits were conducted. The first visit (visit 1) was scheduled at study inclusion after patients had been transferred from ICU to the early neurorehabilitation unit of our hospital. The second visit was conducted at discharge from inpatient care (visit 2). Study visits included comprehensive questionnaires and tests (Table 1). The study visits were predominantly conducted by a physiotherapist (M.Sc.) with 6 year experience in the conduction of clinical trials (ME) or by a medical student after 5 years of medical school (CW).

**Table 1:** Overview of questionnaires and functional tests conducted.

**Type**	**Questionnaires / scales**	**Description**
Fatigue	Fatigue Severity Scale−7 (FSS-7)	This scale evaluates fatigue within seven items. The version with seven items has better psychometric properties compared to the version with nine items (17). Score: 1–7. The cut-off ≥ 4 was interpreted as indicative of fatigue (18).
Anxiety and depression	Hospital Anxiety and Depression Scale (HADS)	This tool is widely used, valid and reliable and repeatedly used in critically ill patients (19, 20). Score: 0–21, each for anxiety and depression. The cutoff value of > 7 indicates the presence of symptoms of anxiety or depression for both subscales (21).
Frailty	Clinical Frailty Scale (CFS)	This scale is reliable and widely used in critical care (22, 23). The revised version with nine items was used (24). Score: 1–9.
Health related quality of life	EQ-5D-5L	This widely used test assesses health-related quality of life (25). Due to higher sensitivity and precision, the version with five answers was used (26). Score:−0.205-1.000.
Dyspnea	Descriptive, visual analog scale (VAS)	Using a VAS from 0 to 10, patients were asked to quantify their severity of dyspnea when walking to the toilet and back (approximately 10 meters). 0 indicates no dyspnea.
Disability/dependence in daily activities	Modified Rankin Scale (mRS)	This clinician-reported, valid and reliable measure of global disability has been widely applied in patients after stroke (27), but it is also used in critically ill patients who are being treated on intensive care units (28, 29). Score: 0–6.
	**Functional test**	**Description**
Functional independence in activities of daily living	Barthel-Index (BI)	This widely used assessment (30) describes the patients' dependence in activities of daily living like washing, grooming, climbing stairs, toilet use etc. It is a reliable and valid tool for patients after critical illness (31). Score 0–100.
Neurological characteristics	Early Rehabilitation Barthel-Index (ERBI)	This reliable and valid extended version of the Barthel-Index containts items like confusion, tracheostomy or dysphagia (32). Score:−325-0.
Olfactory function	Sniffin' sticks	The Sniffin'Sticks screening test with 12 different flavors was used (Burghart Messtechnik, Holm, Germany). The flavors included orange, peppermint, fish, coffee, banana, rose, lemon, pineapple, cinnamon, leather, clove and licorice. Score: 0–12.
Functional mobility (Walking)	Timed-Up-and-Go (TUG)	Functional mobility and balance impairments were assessed with this widely used test which has good psychometric properties (33).
Muscle strength	Grip strength	Grip strength was assessed twice per hand with a digital dynamometer (Kern MAP 130K1, Balingen, Germany). The maximum value was documented.
Walking ability	Functional Ambulation Categories (FAC)	This 6-point scale assesses the ambulation status by determining how much human support the patient requires when walking (34). Score: 0–5.
Basic physical function	Functional Status Score on ICU (FSS-ICU)	This physical function measure was designed for the ICU, comprises five items and has good psychometric properties (35). Score: 0–35.
Cognitive function	Evaluation of attentiveness [Testbatterie zur Aufmerksamkeitsprüfung (TAP)]	This is a computer-based attention test, created by Zimmermann and Fimm (36), which is commonly used in German clinical practice. The TAP consists of the subtests: alertness, Go/NoGo and divided attention.
Cognitive function	Visual and verbal test for retentive-ness [Visueller und Verbaler Merk-fähigkeitstest (VVM)]	Evaluation of retentiveness by using the German test from Schellig and Schächtele (37). Patients have to remember and reproduce visual (a route on a map) and verbal content (information about the building of a museum) within a limited timeframe.

In order to comprehensively determine the patient's clinical course, we retrospectively assessed the preclinical status with regard to preclinical health status, walking ability and frailty. Furthermore, level of education, living and working conditions were recorded. This information was collected during the personal interview of visit 1. Data regarding symptom onset, ICU length of stay, duration of invasive mechanical ventilation, neurological diagnoses as well as pre-existing diseases, Barthel-Index (BI), and Early Rehabilitation Barthel-Index (ERBI) were extracted from patient's health records (32).

Cognitive evaluation was conducted by experienced neuropsychologists (see Table 1). Spirometry was performed to evaluate pulmonary function and to identify restrictive lung diseases.

Medical records were screened for complications and neurological diagnoses, symptoms, and syndromes (e.g., ICUAW, peripheral neuropathy, critical illness polyneuropathy (CIP), critical illness myopathy (CIM), delirium, tetraparesis, dysphagia). The diagnosis ICUAW was defined “as the acute development of generalized weakness in a critically ill patient that cannot be explained by other causes” (38, 39). It encompasses pathologies including critical illness polyneuropathy (CIP), critical illness myopathy (CIM), the combination critical illness neuromyopathy (CINM) and / or muscle atrophy. The diagnose ICUAW based on the medical reports of our or the referring hospital, where the diagnose was set either by the clinical manifestation (weakness, atrophy) or by an electrophysiological investigation. If the patient was transferred from another hospital to ours, we validated the ICUAW diagnose by a clinical investigation of muscle strength (mean strength ≤ 4/5 according to the MRC scale). In inconclusive cases, electrophysiological investigations were conducted (including nerve conduction studies and electromyography were appropriate).

### Statistical analysis

Clinical characteristics are presented as absolute values and percentages, as mean values and standard deviations or as median and interquartile range, as appropriate.

Cognitive impairment was evaluated in percentages according to normative age-dependent values. If the subtest results in the test for attentiveness (TAP) were below 16%, the patient was classified as having limitations concerning attentiveness. If the result of the test for retentiveness (VVM) was below 16%, the patient was classified as having memory and retentiveness limitations. If one result of both test parts was noticeably low, a patient was classified as having general cognitive limitations.

The pulmonary function tests were evaluated to classify the grade of restrictive lung disease. Forced vital capacity (FVC) was set in relation to normative age-dependent values. Percentages ≤ 40% were classified as severe, 41–60% as moderate, 61–80% as light and >80% as no restrictive lung disease. Additionally, FEV1 (forced expiratory volume in 1 s) divided by FVC was used to exclude obstructive lung diseases (with formula values >70%).

For the comparison of symptoms between visit 1 and visit 2 paired *t*-tests were used for interval scaled values, Wilcoxon tests were applied for ordinal scaled values. Friedman-tests with *post-hoc* analysis *via* Dunn-Bonferroni test (including corrected *p*-values) were used for categorical values and comparisons of more than two time points.

A binary logistic regression was calculated to analyze predictors for a high degree of disability and dependence in daily activities (mRS ≥3) at discharge. The independent variables were entered hierarchically: Model 1: age, gender; Model 2: Elixhauser Comorbidity Index, diabetes; Model 3: length of invasive mechanical ventilation. Another hierarchical binary logistic regression model was applied to investigate coefficient predicting whether a subject developed severe fatigue (FSS-7 ≥4 (18)): Model 1: age, gender; Model 2: Elixhauser Comorbidity Index, diabetes; Model 3: length of total hospitalization. A linear multiple regression analysis was used to investigate predictors for hospitalization length. The independent variables were entered hierarchically: Model 1: age, gender; Model 2: Elixhauser Comorbidity Index, diabetes, smoking (within the last 10 years); Model 3: preclinical frailty (CFS).

Statistical analyses were performed using IBM SPSS Statistics 19. A *p* < 0.05 was considered significant. Missing data were not replaced.

## Results

### Study population and disease severity

**Figure 1:**
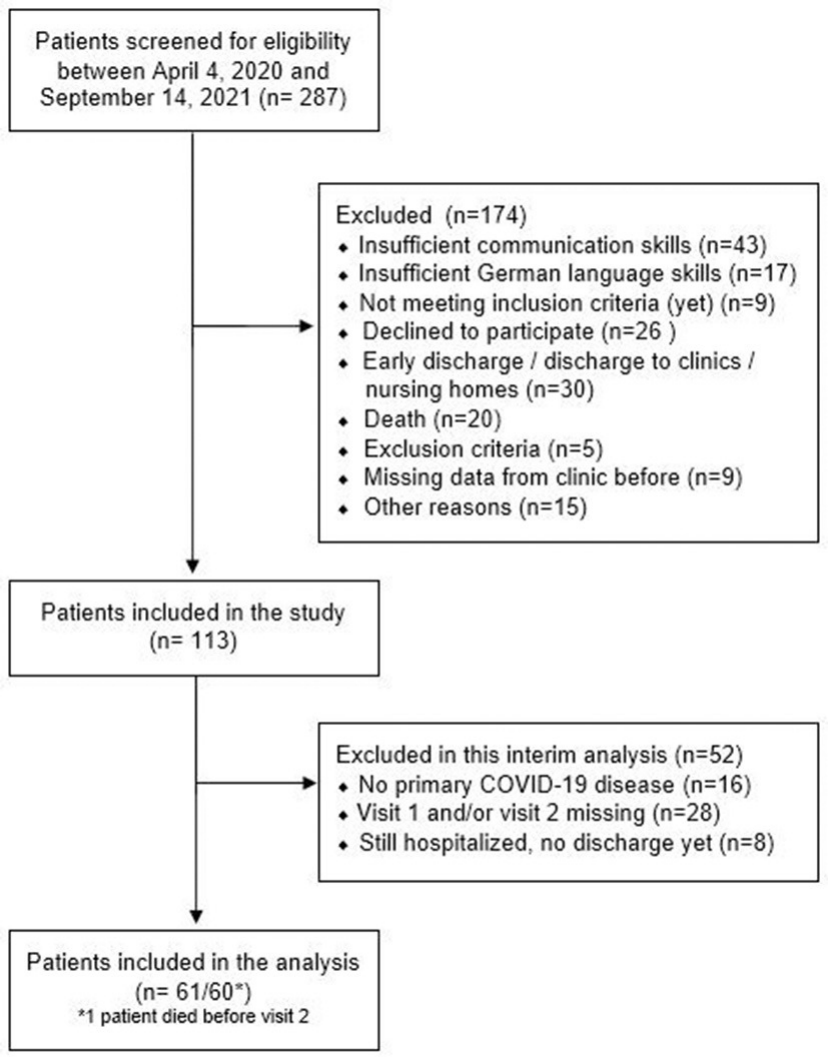
Study flow chart.

Of the 287 patients with COVID-19 admitted to our hospital between April 2020 and September 2021, 113 were enrolled in the study and 61 were included in the current interim analysis. Reasons for exclusion are shown in Figure 1. Patients were included in the study on average 84.6 ± 28.2 days and performed visit 2 on average 120.4 ± 36.9 days after their first positive PCR test.

Patients in our study were profoundly affected by the disease: They overwhelmingly needed long-term critical care therapy (mean ICU length of stay 57.0 ± 22.9 days) with prolonged invasive mechanical ventilation (duration of mechanical ventilation: 45.7 ± 20.7 days) or even extracorporeal membrane oxygenation (ECMO) therapy (16.4%). The length of neurologic rehabilitation after ICU discharge added up to an average of 57.3 ± 26.6 days. Table 2 displays the detailed clinical characteristics of the study population.

**Table 2:** Characteristics of included patients.

	**Total *n* = 61**
Age, years	61.9 ± 12.9, min/max: 38/90
**Sex**	
Women	16 (26.2%)
Men	45 (73.8 %)
Duration of total hospitalization, days	117.8 ± 38.9
Duration of ICU stay, days (*n* = 58)	57.0 ± 22.9
Duration of invasive mechanical ventilation, days (*n* = 57)	45.7 ± 20.7
Duration of inpatient rehabilitation, days	57.3 ± 26.6
Time from first positive PCR-test to study inclusion, days	84.6 ± 28.2
Time from first positive PCR-test to visit 2, days	120.4 ± 36.9
Time from study inclusion to visit 2, days	36.3 ± 23.3, min/max: 8/111
**Highest seven-category scale during hospital stay**	
3: Admitted to hospital, not requiring supplemental oxygen	2 (3.3%)
4: Admitted to hospital, requiring supplemental oxygen	1 (1.6%)
5: Admitted to hospital, requiring HFNC or NIV or both	1 (1.6%)
6: Admitted to hospital, requiring ECMO or IMV, or both	57 (93.4%)
**Complications**	
ARDS	35 (57.4%)
ECMO	10 (16.4%)
Acute kidney failure	20 (32.8%)
Bacterial superinfection	38 (62.3%)
**Cigarette smoking**	
Current smoker	3 (4.9%)
Former smoker	6 (9.8%)
**Comorbidities**	
Diabetes	15 (24.6%)
Obesity	11 (18.0%)
Hypertension	26 (42.6%)
Elixhauser comorbidity index	4.1 ± 7.4, min/max: −7/27
**Education (*****n*** **=** **51)**	
Primary school	2 (3.9%)
Comprehensive school	31 (60.8%)
Grammar school	15 (29.4%)
University	3 (5.9%)
**Occupation before COVID-19**	
Employed	34 (55.7%)
Retired	22 (36.1%)
Volunteer work	3 (4.9%)
Unemployed	2 (3.3%)
**Living conditions**	
At home alone	11 (18.0%)
At home not alone (e.g., with family)	49 (80.3%)
Nursing home	1 (1.6%)
**Preclinical status**	
Frailty (CFS)	2 (1)
Overalls disability (mRS)	0 (0)
Functional independence (Barthel-Index)	100 (0)
Walking ability (FAC)	5 (0)

Data are n (%), mean ± SD or median (interquartilerange).

HFNC, high-flow nasal cannula for oxygen therapy; NIV, non-invasive ventilation; IMV, invasive mechanical ventilation; ICU, intensive care unit; ECMO, extracorporeal membrane oxygenation; BMI, Body Mass Index: normal weight = BMI < 25 and obesity = BMI ≧ 25; Never smoker, never smoked at all or quit smoking more than 10 years ago. CFS, Clinical Frailty Scale; mRS, modified Rankin Scale; FAC, Functional Ambulation Categories.

### Pulmonary dysfunction

Forty-seven patients underwent a lung function test at study inclusion (87.5 ± 31.4 days after the first positive PCR test), of which four had to be excluded due to a lack of cooperation. Of the remaining 43, only two showed signs of obstructive lung disease, whereas most patients (*n* = 31; 72.1%) were diagnosed with restrictive lung disease of varying severity [light: *n* = 16 (37.0%); moderate: *n* = 12 (27.9%); severe: *n* = 3 (7%)]. Only 10 patients (23.2%) displayed a normal lung function test.

### Neurological disorders and cognitive impairment after severe COVID infection

All patients had severe neurological deficits requiring intense neurological rehabilitation. ICUAW (CIP/CIM) was the hallmark diagnosis (*n* = 56; 91.8%), followed by delirium in 19 (31.1%), and encephalopathy in 11 patients (18.0%). Other neurological diagnoses were cerebral ischemia (*n* = 5; 8.2%), epileptic seizures (*n* = 4; 6.6%), and Guillain-Barré-Syndrome (*n* = 2; 3.3%).

In accordance with the high prevalence of CIP/CIM, most patients suffered from incomplete tetraparesis. Other common symptoms were dysphagia (*n* = 28; 45.9%), hypoesthesia/paresthesia/neuropathic pain (*n* = 9; 14.8%), paresis (*n* = 7; 11.5%; hemiparesis, facial palsy or monoparesis due to peripheral nerve lesions), and tremor (*n* = 2; 3.3%).

At study inclusion, 47 participants underwent cognitive testing, with *n* = 36 (76.6%) showing cognitive impairments. Deficits in the memory and retentiveness component were apparent in *n* = 26 of 44 participants (59.0%). Regarding the attentiveness component (conducted in 46), *n* = 26 (56.6%) showed deficits. Problems in both components were apparent in *n* = 16 (37.2%, *n* = 43 completed both tests). The 14 remaining patients were either not able to perform the tasks of cognitive testing due to language barriers or due to an insufficient functional ability to use the computer or to hold a pencil.

### Clinical course and health status at discharge

**Figure 2:**
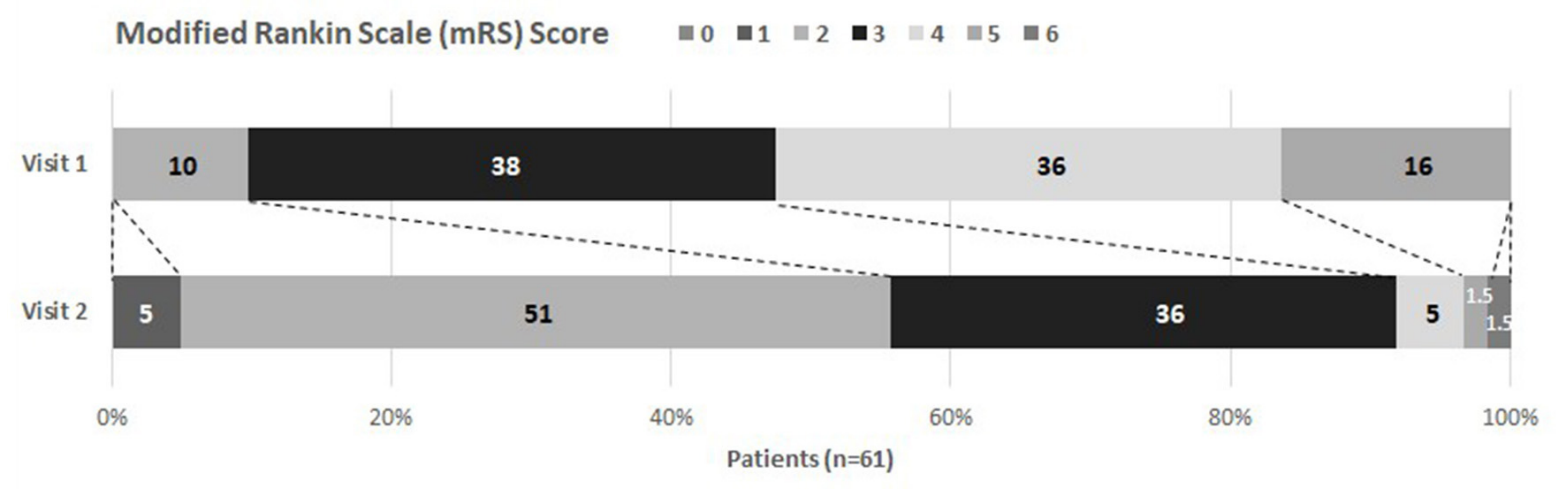
Modified Rankin Scale at visit 1 and 2. Visit 1 took place at study inclusion, visit 2 at discharge from inpatient care. Percentages of each category are given. A shift of mRS values can be noticed (depicted with dotted lines) showing improvement between visits.

Results of assessments and questionnaires are shown in Table 3. From study inclusion to discharge, patient's health status significantly improved in all measured categories except for fatigue and anxiety. Figure 2 illustrates this for the mRS, where most of the patients improved their status [at admission most patients were classified into category 3 (38%) or 4 (36%), whereas at discharge most patients improved to category 2 (51%) and 3 (36%)]. However, health status and body function remained substantially reduced at discharge, represented by reduced mobility, restricted independence in activities of daily living, muscle strength, and breathing. Furthermore, impairments were observed for the olfactory sense with 37/56 (66.1%) being impaired, and pain/ discomfort with 45/60 (75%) being affected. Altogether, patients' health-related quality of life improved significantly during neurorehabilitation, but was still impaired at discharge.

**Table 3:** Results of the questionnaires and functional tests.

	**At study inclusion**	**At discharge**	***p*-values**	**Z / T / χ2**	**Effect size r / Cramer's V**
mRS	4.0 (1.0)	2.0 (1.0)	*p* < 0.001	Z = −5.675	r = −0.514
FAC	3.0 (2.75)	5.0 (1.0)	*p* < 0.001	Z = −6.491	r = −0.593
CFS	6.0 (1.75)	5.0 (2.0)	*p* < 0.001	Z = −5.656	r = −0.516
FSS-7 Fatigue ≥ 4	2.8 (2.8) 17 (28.3%)	2.9 (2.6) 13 (22.1%)	*p* = 0.970 *p* = 0.429	Z = −0.038 χ2 = 0.626	r = −0.003 φ. = 0.73
Grip Strength max. [kg]	17.1 ± 6.7	20.3 ± 7.9	*p* < 0.001	T = −5.645	r = 0.720
Barthel-Index Early rehabilitation Barthel-Index	42.5 (35.0) 0.0 (−100.0)	65.0 (7.5) 0.0 (0.0)	*p* < 0.001 *p* < 0.001	Z = −6.477 Z = −4.163	r = −0.591 r = −0.380
FSS-ICU	30.0 (7.75)	34.0 (2.0)	*p* < 0.001	Z = −6.381	r = −0.583
**HADS**					
Anxiety Anxiety > 7 Depression Depression > 7	5.0 (5.8) 21/61 (34.4%) 4.0 (5.0) 15/61 (24.6%)	4.0 (4.0) 11/59 (18.6%) 3.0 (5.0) 8/59 (13.6%)	*p* = 0.142 *p* = 0.062 *p* = 0.026 *p* = 0.170	Z = −3.385 χ2 = 4.048 Z = −2.810 χ2 = 1.886	r = −0.312 φ. = 0.184 r = −0.259 φ. = 0.126
Sniffin' Sticks Normal (11–12) Hyposmia (7–10) Anosmia (1–6)	8.6 ± 2.3 14/59 (23.7%) 37/59 (62.7%) 8/59 (13.6%)	9.2 ± 2.4 19/56 (33.9%) 31/56 (55.4%) 6/56 (10.7%)	*p* = 0.003 *p* = 0.473	T = −3.057 χ2 = 1.495	r = 0.384 φ. = 0.114
TUG [s] Unable to walk Walking aid required (of those who were able to walk)	22.6 ± 17.2 14/58 (24.1%) 23/44 (52.3%)	16.4 ± 16.3 1/56 (1.8%) 13/55 (23.6%)	*p* = 0.001 *p* < 0.001 *p* = 0.003	T = 3.498 χ2 = 12.458 χ2 = 8.663	r = 0.489 φ. = 0.331 φ. = 0.296
**EQ-5D-5L**					
Health Scale Index value Problems with walking around Problems with washing or dressing Problems with usual activity Pain or discomfort Anxiety or depression	52.3 ± 18.0 0.554 ± 0.287 58/60 (96.7%) 49/60 (81.7%) 49/60 (81.7%) 48/60 (80.0%) 32/60 (53.3%)	67.4 ± 16.6 0.749 ± 0.176 45/59 (76.3%) 32/59 (54.2%) 32/59 (54.2%) 45/59 (76.3%) 15/59 (25.4%)	*p* < 0.001 *p* < 0.001 *p* = 0.001 *p* = 0.001 *p* = 0.001 *p* = 0.623 *p* = 0.002	T = −5.730 T = −5.877 χ2 = 10.633 χ2 = 10.297 χ2 = 10.297 χ2 = 0.242 χ2 = 9.697	r = 0.601 r = 0.608 φ. = 0.299 φ. = 0.294 φ. = 0.294 φ. = 0.045 φ. = 0.285
Dyspnea	4.3 ± 2.6	3.3 ± 2.6	*p* = 0.035	T = 2.156	r = 0.277

Data are n/N (%), mean ± SD or median (Interquartilerange).

mRS, modified Rankin Scale; FAC, Functional Ambulation Categories; CFS, Clinical Frailty Scale; FSS-7, Fatigue-Severity-Scale-7; FSS-ICU, Functional Status Score on ICU; HADS, Hospital Anxiety and Depression Scale; TUG, Timed-Up-and-Go. The effect size r was calculated with $r=z/\sqrt{N}$ for ordinal scaled values and with $r=\sqrt{\frac{t^{2}}{t^{2}+df}}$ for metric scaled values and with Cramer's V (calculation by SPSS) for categorical values.

**Figure 3:**
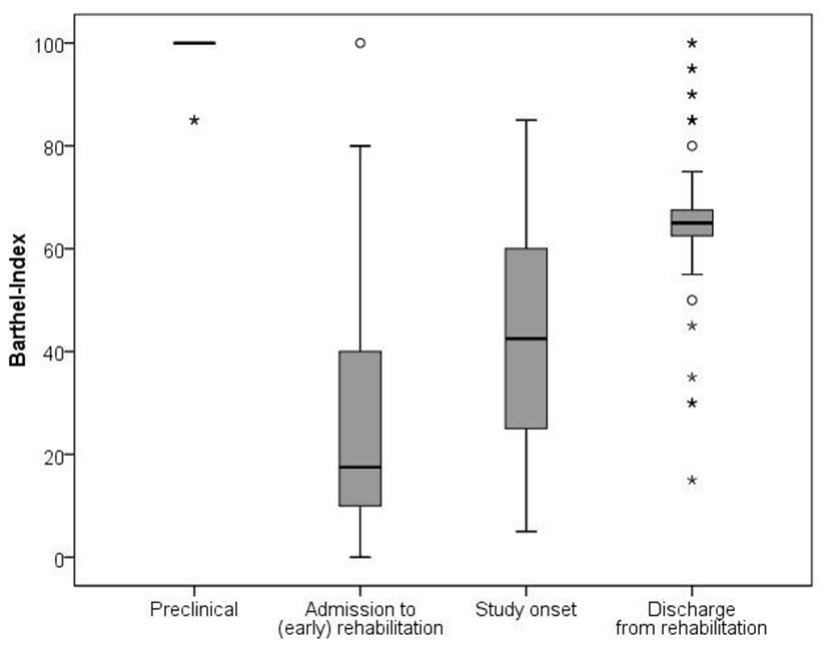
Development of the Barthel Index from preclinical state throughout rehabilitation. The comparison between the four time points showed significant differences and large effect sizes (Friedman-test and effect size with r = z/√N): preclinical-hospital admission (*p* < 0.001, r = −0.612); preclinical-study onset (*p* < 0.001, r = −0.616); preclinical-hospital discharge (*p* < 0.001, r = −0.615); hospital admission-study onset (*p* = 0.035; r = −0.487); hospital admission-hospital discharge (*p* < 0.001, r = −0.600); study onset-hospital discharge (*p* < 0.001, r = −0.591).

Figure 3 shows a significant improvement of the BI from initial admission (median 17.5; IQR 30.0) *via* visit 1 (median 42.5; IQR 35.0) to visit 2 (median 65.0; IQR 7.5) [χ2(3) = 167.7; *p* < 0.001]. Notably, there was still a significant gap regarding the BI between the patients' condition at hospital discharge and their preclinical condition (median 100.0; IQR 0.0, *p* < 0.001). *Post-hoc* analysis revealed significant differences between all measurements of the BI.

**Figure 4:**
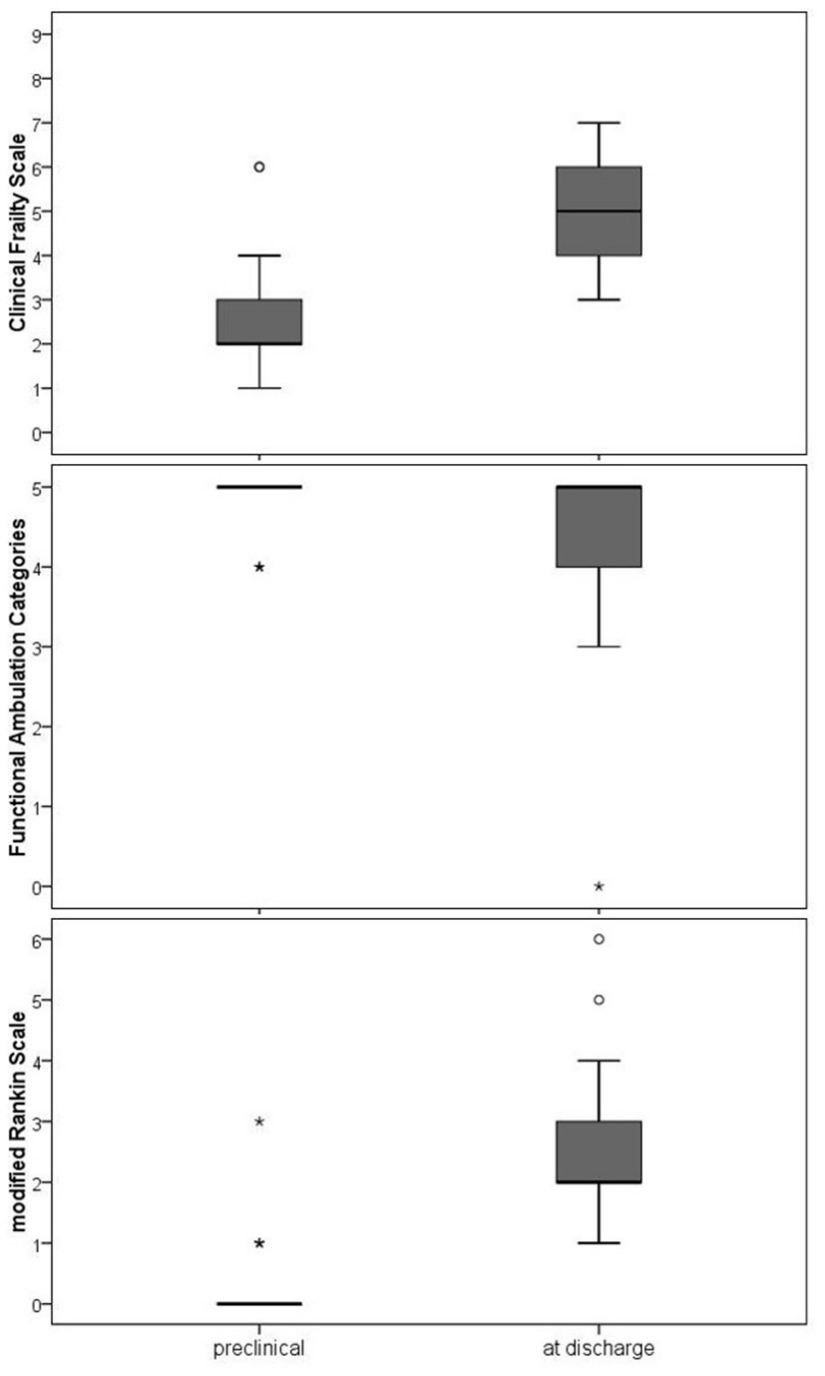
Walking ability, frailty and overall disability at clinical discharge compared to the preclinical condition. FAC (Functional Ambulation Categories), Frailty (Clinical Frailty Scale), mRS (modified Rankin Scale). The Wilcoxon test was used to compare the preclinical state and the state at discharge. All three comparisons differed statistically significant (*p* < 0.001).

As shown in Figure 4, the condition of the patients was still limited at hospital discharge in comparison to the preclinical state. The FAC showed complete independence in preclinical walking but a significantly restricted ability at discharge (Z = −3.862, *p* < 0.001). As presented with the CFS, patients did not achieve their preclinical state of frailty (Z = −6.570, *p* < 0.001). The mRS underlines the overall significantly impaired health state at discharge (Z = −6.885, *p* < 0.001).

Forty-four patients (73.3%) were discharged to their homes, 14 (23.3%) were discharged to another rehabilitation facility (not to hospitals), and 2 (3.3%) returned to their nursing home and assisted living facility. Among those participants employed before disease onset, all were discharged as incapable of working.

### Predictors for disability, fatigue and hospitalization length

Regression analyses did not result in significant models (*p* > 0.18); none of the coefficients was found to be predictive for the disability at discharge, fatigue at discharge, or the length of hospitalization (*p* > 0.10).

### Medical and assistive devices

At study inclusion, 15 patients (25.0%) needed oxygen, 15 (25%) had a permanent bladder catheter, 13 (21.7%) required a tracheal tube, 4 (6.7%) had a percutaneous endoscopic gastrostomy, 3 (5.0%) needed an ileostomy, 2 (3.3%) had a nasogastric tube, and 1 (1.7%) required negative pressure wound therapy.

At discharge, 7 participants (11.7%) still needed oxygen, 6 (10%) had a permanent bladder catheter, and 2 (3.3%) needed an ileostomy.

At discharge, the majority of participants still needed assistive devices for their activities of daily living. Only 14 participants (23.3%) did not require any aids and appliances. Most frequently used were walker-rollators [33 (55.0%)], wheelchairs [16 (26.7%)], toilet and shower chairs [14 (23.3%)], ankle-foot orthosis [7 (11.7%)], nursing beds [6 (10.0%)], bathroom handles [6 (10.0%)] and oxygen concentrators [5 (8.3%)].

## Discussion

We here report on the clinical course in a cohort of the most severely affected patients with COVID-19 disease who required long-term inpatient care and rehabilitation because of neurological deficits. After the ICU-treatment, patients showed mainly muscular weakness (ICUAW, CIP/CIM) and cognitive deficits (delirium, encephalopathy). Our main findings are: (1) Patients with critical COVID-19 spent on average 4 months in hospital; most of them showed relevant muscular weakness due to ICUAW. (2) Patients improved over time, but still suffered from substantial deficits at discharge. Patients in general did not reach the preclinical health status, as indicated by the overall disability (mRS) and frailty (CFS). (3) We could not identify any predictors for the degree of disability and fatigue at discharge or for the length of hospitalization.

Previous studies reported on benefits and effectiveness of rehabilitation in patients after COVID-19. However, these studies focused on less severely affected patients, shorter rehabilitation periods, earlier rehabilitation phases, less intensive rehabilitation, or other organ systems (e.g., pulmonary rehabilitation) (11, 12, 40, 41).

Despite the long duration of rehabilitation and total hospitalization in our cohort, the functional and health status at discharge was worse compared to other studies (12, 40, 41). This most likely reflects the prolonged treatment on ICU with mechanical ventilation in our sample. The maximum number of days on ICU reported before were 18–22 days (40, 42, 43).

The impaired health status at discharge is clearly represented by the gap in mRS and the CFS between the preclinical state and the state at discharge. Both assessments, as well as the BI, indicate the patients' need for help in nearly all their activities of daily living (ADL). This clearly affects their level of independence. Limitations in ADLs after the acute phase of COVID-19 were previously reported (44). Our results show that ADL limitations after critical COVID-19 disease last for a prolonged period of time. Furthermore, health-related quality of life was still substantially impaired compared to a sample of healthy German seniors, although we found a significant improvement over time (45). Our values in this domain were also lower than those reported in a study on 47 patients after COVID-19 who required mechanical ventilation for a median of 12 days. In that study, the VAS in the EQ-5D-5L was 80 (70–90) 3 months after hospital admission and only 40% reported problems in the dimension of mobility (compared to 75% in our cohort) (46). This difference again can be explained by the critical course of disease with a prolonged length of hospitalization in our cohort. Our EQ-5D-5L results are in a similar range as reported for (1) patients with chronic conditions like cardiovascular disease or depression (VAS: 64±23) (47) and (2) general critical illness survivors with a median length of 10 days for mechanical ventilation [e.g., VAS: 64 ± 23; index: 0.73 (IQR = 0.3)] (48). However, those patients reported higher values of anxiety (as measured by the HADS; median = 7.0) compared to our patients (median = 4.0). This might be explained by feelings of relief and gratefulness for surviving COVID-19, which were frequently mentioned by our participants. In contrast, reported depression values were quite similar to the results of our study (48). Our values for anxiety and depression are in accordance with HADS values reported in a cohort ~4 months after hospital admission due to COVID-19 (median duration of mechanical ventilation: 19 days) (42).

Regarding fatigue, we expected higher values in our group of critically ill patients, similar to reported values in several studies on patients post-COVID (4). In a meta-analysis on hospitalized patients, 38.4% suffered from fatigue (95% CI 30.4–47.4) over 90 days after symptom onset (49). The mild fatigue score in our group (noticeable in only ~25%) might be explained by the fact that our patients were still in a hospital setup without the challenging responsibilities, duties or long-lasting physical activities required for ADL.

Our results show that even after an average of >100 days of hospitalization including >50 days of intensive neurorehabilitation, the health state and functional capacity after severe COVID-19 disease is limited. Therefore, a long-term disability can be anticipated, especially when considering the sequelae reported in less severely affected patients. Huang et al. (50) reported in a trial on 1,733 hospitalized patients post COVID-19 that 76% experienced at least one symptom like fatigue or muscle weakness, sleep difficulties, anxiety and depression 6 months after infection. This percentage increased to 86% in a subgroup of patients who needed (non-)invasive ventilation (50). Within a cohort of 246 ICU survivors after critical COVID-19, Heesakkers et al. (43) reported than 74.3% experienced physical symptoms, 26.2% experienced mental symptoms and 16.2% experienced cognitive symptoms even 1 year after ICU treatment (43). Therefore, further evaluations of symptoms and their impact on activity and participation in daily life are urgently needed.

Our study has some limitations. First, a non-COVID-19 control group with similar motor and cognitive deficits would have been of value to compare outcomes. It is not clear how specific our findings are for COVID-19. Furthermore, evaluation of lung function, cognitive impairment and nerve conduction studies were only conducted once. Thus, no assertions can be made about their potential improvement during rehabilitation. Additionally, a high number of screened patients were not included in the study (226 of 287) mainly due to insufficient communication skills. Furthermore, 19 patients were excluded, because we were only able to conduct one study visit due to a rapid discharge (~ 1–5 days) after the first study visit. We did an analysis to investigate the characteristics of those 19 excluded patients compared to the 61 included patients. Patients who were excluded were significantly younger (mean age 54.3 vs. 61.9 years), had significantly less comorbidities, were significantly shorter on ICU (44.1 vs. 57.0 days), had significantly less days of mechanical ventilation (26.9 vs. 45.7 days) and had a significantly shorter duration of complete hospitalization (78.6 cs. 119.4 days). However, both patient groups did not significantly differ in their health status at discharge regarding any assessment (e.g., Barthel Index, HADS, EQ-5D-5L, mRS, CFS, FSS-ICU, FAC, FSS-7). It can be concluded, that younger patients with a better preclinical health status required less intensive care and recovered faster. However, their health status at discharge was as limited as the health status of patients with a longer hospitalization period and a worse preclinical health status. Finally, single-center studies bear the risk of bias, which for example becomes apparent as our center included only critically affected patients.

In summary, our findings stress the need for intensive neurorehabilitation in patients with severe neurological symptoms after critical COVID-19 disease. We cannot determine the specific effect of rehabilitation. However, deficits are pronounced and do not resolve on a short time scale. We report substantial and long-lasting limitations regarding the general health status, dependence in ADL and health related quality of life even at discharge. As persistent limitations after critical COVID-19 disease are a socioeconomic and medical challenge, further research characterizing the neurological aspects of the pandemic disease and developing tailored rehabilitation and home care programs is of paramount interest.

## Data availability statement

The raw data supporting the conclusions of this article will be made available by the authors, without undue reservation.

## Ethics statement

The studies involving human participants were reviewed and approved by the Ethical Committee of the Medical Faculty of the Ludwig-Maximilians University of Munich. The patients/participants provided their written informed consent to participate in this study.

## Author contributions

CW: data curation (equal), formal analysis (equal), investigation (equal), resources (support), visualization (equal), writing—original draft preparation (equal), and writing—review and editing (equal). ME: conceptualization (equal), data curation (equal), formal analysis (equal), funding acquisition (support), investigation (lead), methodology (lead), project administration (support), resources (lead), validation (equal), visualization (equal), writing—original draft preparation (equal), and writing—review and editing (equal). JB: formal analysis (equal), funding acquisition (lead), methodology (support), project administration (lead), validation (equal), and writing—review and editing (equal). VH: validation (equal), resources (support), writing—original draft (support), and writing—review and editing (equal). FM: conceptualization (equal), methodology (support), supervision (support), validation (equal), and writing—review and editing (equal). KJ: conceptualization (equal), funding acquisition (support), methodology (support), project administration (lead), supervision (lead), validation (equal), visualization (support), writing—original draft preparation (equal), and writing—review and editing (equal). All authors contributed to the article and approved the submitted version.

## Funding

This work was partly funded by the Else Kröner-Fresenius-Stiftung (2021_EKEA.78).

## Conflict of interest

The authors declare that the research was conducted in the absence of any commercial or financial relationships that could be construed as a potential conflict of interest.

## Publisher's note

All claims expressed in this article are solely those of the authors and do not necessarily represent those of their affiliated organizations, or those of the publisher, the editors and the reviewers. Any product that may be evaluated in this article, or claim that may be made by its manufacturer, is not guaranteed or endorsed by the publisher.
